# Mineral Soils Are an Important Intermediate Storage Pool of Black Carbon in Fennoscandian Boreal Forests

**DOI:** 10.1029/2022GB007489

**Published:** 2022-11-16

**Authors:** Johan A. Eckdahl, Pere Casal Rodriguez, Jeppe A. Kristensen, Daniel B. Metcalfe, Karl Ljung

**Affiliations:** ^1^ Department of Physical Geography and Ecosystem Science Lund University Lund Sweden; ^2^ Department of Ecology and Environmental Science Umeå University Umeå Sweden; ^3^ Department of Geology Lund University Lund Sweden; ^4^ Environmental Change Institute School of Geography and the Environment University of Oxford Oxford UK

**Keywords:** black carbon, pyrogenic carbon, boreal wildfire, carbon cycle, mineral soil, fire severity

## Abstract

Approximately 40% of earth's carbon (C) stored in land vegetation and soil is within the boreal region. This large C pool is subjected to substantial removals and transformations during periodic wildfire. Fire‐altered C, commonly known as pyrogenic carbon (PyC), plays a significant role in forest ecosystem functioning and composes a considerable fraction of C transport to limnic and oceanic sediments. While PyC stores are beginning to be quantified globally, knowledge is lacking regarding the drivers of their production and transport across ecosystems. This study used the chemo‐thermal oxidation at 375°C (CTO‐375) method to isolate a particularly refractory subset of PyC compounds, here called black carbon (BC), finding an average increase of 11.6 g BC m^−2^ at 1 year postfire in 50 separate wildfires occurring in Sweden during 2018. These increases could not be linked to proposed drivers, however BC storage in 50 additional nearby unburnt soils related strongly to soil mass while its proportion of the larger C pool related negatively to soil C:N. Fire approximately doubled BC stocks in the mineral layer but had no significant effect on BC in the organic layer where it was likely produced. Suppressed decomposition rates and low heating during fire in mineral subsoil relative to upper layers suggests potential removals of the doubled mineral layer BC are more likely transported out of the soil system than degraded in situ. Therefore, mineral soils are suggested to be an important storage pool for BC that can buffer short‐term (production in fire) and long‐term (cross‐ecosystem transport) BC cycling.

## Key Points

- Fifty separate summer 2018 boreal wildfires were sampled 1 year postfire for black carbon (BC) across the 57–67° latitudinal range within Sweden
- Fire had an insignificant effect on total BC stocks in organic layers but approximately doubled stocks in mineral layers
- Mineral soil may function to buffer short‐term (production) and long‐term (cross‐ecosystem transport) BC processes

## 1 Introduction

The boreal landscape stores an estimated 40% of the 2,500 Pg carbon (C) found in the earth's terrestrial vegetation and soils (Bradshaw & Warkentin, 2015; Friedlingstein et al., 2020). Presently, these stores are sequestering atmospheric C, helping to offset human emissions of greenhouse gasses (Lemprière et al., 2013; Tagesson et al., 2020). The most rapid transfer of C from forested boreal regions to the atmosphere occurs during wildfires which ignite sporadically during periods of extremely hot and dry weather (Bond‐Lamberty et al., 2007; Schultz et al., 2008) and release an estimated 198 Tg C to the atmosphere yearly (van der Werf et al., 2017). Increases in wildfire activity in the 21st century threaten to slow or reverse the present trend of C sequestration across the boreal region, further accelerating increases of atmospheric CO_2_ and climate change (de Groot et al., 2003; de Groot, Flannigan, & Cantin, 2013; Gillett et al., 2004; Kelly et al., 2013; Li et al., 2017).

Although wildfires immediately release a substantial portion of C to the atmosphere, a fraction of heat‐altered C is left behind in the soil as pyrogenic carbon (PyC) (Coppola et al., 2022; Jones et al., 2019; Santín et al., 2015). In addition to being a substrate for respiration, PyC has many influences on the soil environment which affect overall C cycling and other ecosystem processes. In particular, freshly produced PyC can have substantially greater specific surface area and ion exchange capacity than its parent material allowing it to better immobilize otherwise leachable, fire‐mineralized nutrients, releasing them gradually (Makoto et al., 2011, 2012; Warnock et al., 2007). PyC can also improve soil water retention, soil aeration, and provide microbial habitat structure (Warnock et al., 2007). However, these effects vary depending on the properties of PyC introduced to the ecosystem. Differing parent material along with varied levels of heating and concurrent oxygen availability during a fire event result in the production of a broad spectrum of different PyC structures (McBeath et al., 2011; Preston & Schmidt, 2006; Schmidt & Noack, 2000; Wiedemeier et al., 2015). This great variety of PyC material, and the range of environmental conditions in which it is found, has resulted in mixed views on its overall susceptibility to remineralization, mobility within the soil profile and role in soil biogeochemical cycling (Bird et al., 2015; Bowring et al., 2022; Coppola et al., 2022; Santín et al., 2016).

Once PyC is added to the soil through burning, physical displacement can alter its susceptibility to remineralization. For example, downward displacement by bioturbation or percolating water can remove PyC from the most biologically active topsoil regions and exposure to high heat during repeated fire (Bellè et al., 2021; Czimczik & Masiello, 2007). However, boreal forests have relatively slow vertical mixing mechanisms and so within these ecosystems PyC production and storage is thought to be restricted to organic surface layers where it has increased vulnerability to remineralization (Czimczik & Masiello, 2007; Preston & Schmidt, 2006; Santín et al., 2015). Additionally, lateral displacement allows PyC to enter waterways where a portion can be deposited as sediment in lakes and rivers (Coppola et al., 2022; Jones et al., 2020). Since it is generally more resistant to degradation than non‐pyrogenic C, PyC has a greater chance of surviving further transport to ocean sediments, thereby drawing attention to the role of wildfire in connecting the fast (biosphere‐atmosphere) and slow (geological) C cycles (Coppola et al., 2022; Jones et al., 2020) which can account for missing sinks within the global C cycle (Coppola et al., 2022; Santín et al., 2015). Despite total burnt area in boreal catchments being concentrated within fire seasons separated by several years, downstream ratios of dissolved PyC to total dissolved organic C appear to be consistent over time, suggesting extended PyC dissolution mechanisms which may be shared with the non‐pyrogenic organic soil C pool (Jaffé et al., 2013; Jones et al., 2020; Wagner et al., 2015). Therefore, determining the relative quantity and location of PyC within the larger soil C pools where it is stored is a crucial first step in understanding its transport and degradation mechanisms.

Global quantification of the PyC cycle using published data has proven difficult largely due to the wide variety of sampling and characterization methods employed that capture different fractions of the PyC continuum (Hammes et al., 2007; Reisser et al., 2016). While PyC inputs (production due to fire) and outputs (storage in ocean sediments) to and from the fast C cycle are beginning to be quantified globally, detailed knowledge regarding intermediate storage pools and transport mechanisms is severely lacking (Coppola et al., 2022; Jones et al., 2019; Reisser et al., 2016; Santín et al., 2016). In particular, little is known about the drivers of PyC addition to soils due to wildfire within and across landscape types and how its immediate production and removal is distributed among soil layers. Further, the rate of addition of PyC to forest ecosystems has been observed to far outweigh the presumed low within‐soil decomposition rates, suggesting extensive physical displacement between fire events that is currently not well understood (Bowring et al., 2022; Coppola et al., 2022; Santín et al., 2016). As a result, the large variation in PyC production and stocks due to varied combustion conditions and time since fire documented in the literature present challenges to the development of predictive knowledge of BC production and transport that accounts for the diversity of fire conditions and ecosystem structure in which they occur (Jones et al., 2019; Reisser et al., 2016; Santín et al., 2016). It is therefore of additional importance when constraining estimates of total PyC across ecosystems to identify predictive, broad scale environmental and fire‐associated variables that better link small‐scale BC cycling knowledge to the landscape level.

The currently most utilized variable for predicting PyC production in response to wildfire across landscapes are upscaled estimates of wildfire C emissions, which can be directly multiplied by regionally assigned PyC production factors, derived from published observations, to estimate total PyC production within a given burnt area (Jones et al., 2019). These production factors are designed to reflect variation in vegetation and surface fuel structure and lack validation especially in boreal ground fires, where smoldering can occur deep within thick organic soil layers. These factors also do not consider the additional effects of the extent of fuel drying during a fire season which are determined by soil drainage conditions, evapotranspiration and moisture input deficits (Grant et al., 2009). These properties may potentially alter the completion of the combustion reaction, where moisture can act as a heat sink and inhibit oxygen availability around fuel sources, especially in porous soil environments where gas exchange with the atmosphere is limited (Frandsen, 1987). Climate conditions can also control ground fuel structure and thus its susceptibility to desiccation and combustion (Eckdahl, Kristensen, & Metcalfe, 2022; Hanan et al., 2022). Longer term, postfire storage of PyC in soils is thought to be controlled in large part by its resistance to decay (Bowring et al., 2022; Coppola et al., 2022) and therefore its amount relative to the entire soil C pool (i.e., BC:C) is expected to increase in more advanced stages of decomposition of the soil compartment in which it is stored, as measured by reduced soil C to nitrogen ratio (C:N) (Callesen et al., 2007). Additionally, the more mobile components of PyC (e.g., those which are found in waterways) may require adsorption to stationary soil material in order to remain within the soil profile over longer periods and hence the amount of this PyC fraction may correlate with total soil mass with additional influences of soil drainage and precipitation. Though these trends can be intuitively supported by existing knowledge regarding combustion conditions and PyC properties, they have limited observational support across larger regions and gradients of wildfire conditions, especially within ground‐fire dominated boreal forests (Reisser et al., 2016).

This study aimed to address these research gaps by using an established network of 50 Fennoscandian boreal forest wildfires (all occurring in summer 2018 across Sweden) that demonstrated the linkage of climate to fuel loading and structure and its resulting combustibility as measured by ecosystem C loss and soil charring (Eckdahl, Kristensen, & Metcalfe, 2022). The 50 wildfire burnt plots of the study were each paired with a nearby, unburnt control plot to survey PyC throughout the soil profile at the 1 year postfire stage across gradients of climate, soil drainage, soil total C, soil C:N, fire severity (C loss) and time‐of‐fire drought conditions. Analyzing a particularly chemically inert portion of the PyC continuum, here called black carbon (BC), and its proportional weight in the total C pool (i.e., BC:C), it was hypothesized that:

1. Fire increased total BC and BC:C in both organic and mineral soil layers.
2. Fire‐induced changes of total BC and BC:C in soils were related to climate, time‐of‐fire drought conditions, soil drainage and wildfire C loss.
3. Total BC and BC:C stored in sampled control plot soils were related to climate as well as their mass, C, C:N, and drainage conditions.

## 2 Materials and Methods

### 2.1 Plot Selection

**Figure 1:**
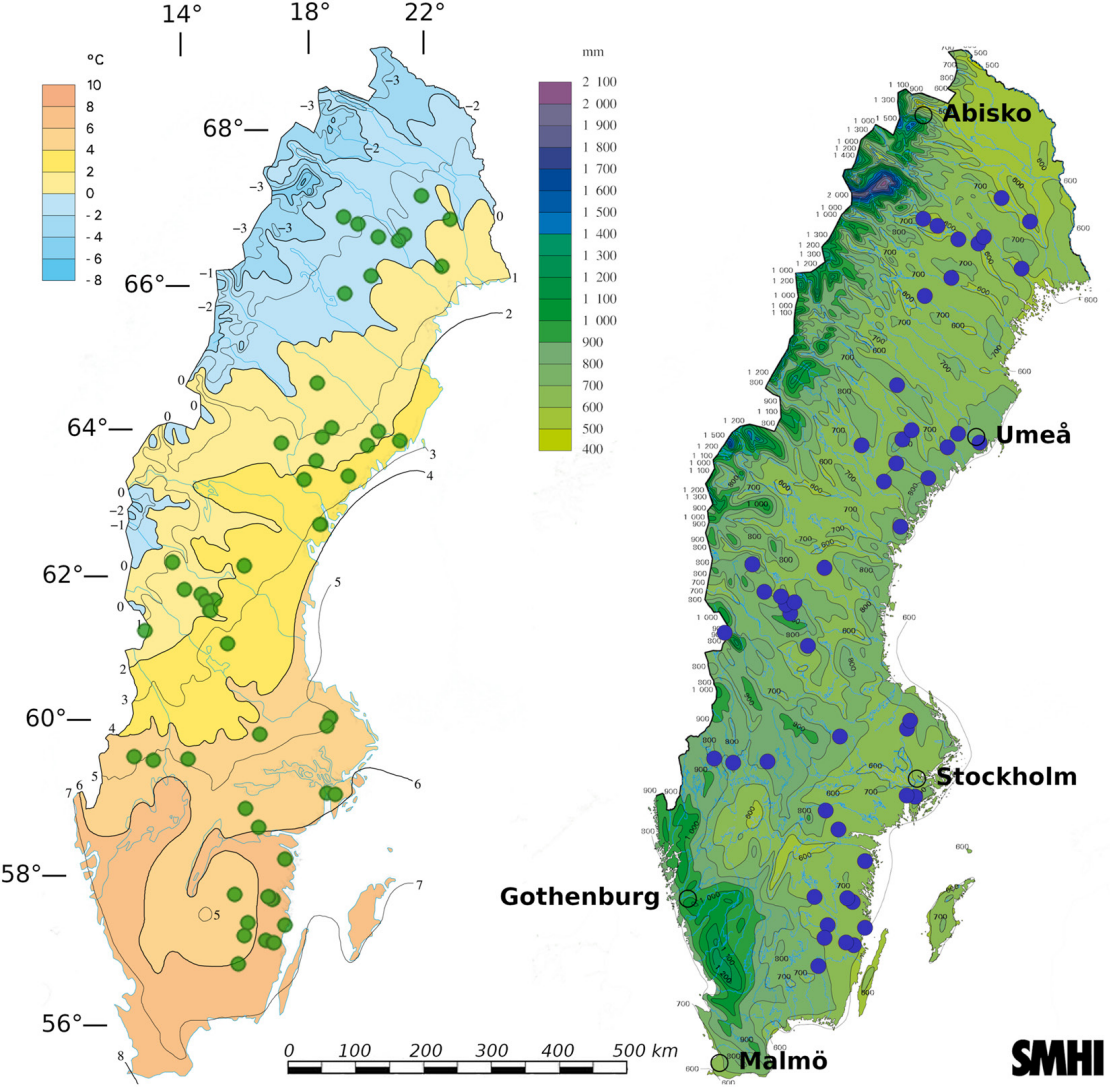
The 50 plot pairs spanned the approximately 57–67° latitudinal range within Sweden. They are seen as colored points on figures provided by the Swedish Meteorological and Hydrological Institute of mean annual temperature (left) and mean annual precipitation (right) over the last normal period 1961–1990. Major cities/research centers are marked with empty circle perimeters and WGS 84 (EPSG:4979) latitudes (horizontal bars) and latitudes (vertical bars) are given in degrees.

This study utilized 50 separate wildfires occurring during summer 2018 in Sweden, which were part of a previous published study linking climate variation to fire severity (Eckdahl, Kristensen, & Metcalfe, 2022). The 50 burnt plots were selected to maximize spread across climate gradients in Sweden from a pool of 325 fires identified during the summer 2018 period that had perimeters manually mapped by the Swedish Forest Agency. Perimeters were drawn around burn scars identified using Normalized Burn Ratio (NBR) values derived from Sentinel‐2 bottom‐of‐atmosphere corrected bands 8 and 12. Close to the highest NBR pixel values in each separate burn scar, one 20 × 20 m^2^ plot was established along with an equally sized, paired control plot located in unburnt forest between 15 and 150 m (average 58 m) outside the burn scar. The control plots served as estimates of prefire properties for each of their corresponding burnt plots. Using geodatasets prior to the field campaign, plot pairs were best matched to resemble each other in terms of overstory biomass, basal area, tree species dominance, and stand age. Topo‐edaphically evaluated soil moisture potential (TEM), which is considered a metric of soil drainage, was also used to match plots and avoid wetland areas (Naturvårdsverket, 2018). TEM was provided at 10 m resolution and given as integer values ranging from 0 to 240 (in order of increasing moisture potential) and was based on the Soil Topographic Wetness Index (Buchanan et al., 2014) in areas where soil type information was available and on the two topographic indices Depth to Water (Murphy et al., 2007) and the Topographic Wetness Index (Beven & Kirkby, 1979) where soil information was unavailable. Elevation data was provided by the Swedish Mapping, Cadastral and Land Registration Authority from a 50 m resolution digital elevation model (Lantmäteriet, 2021). Slope was calculated using the “slope” function within the ArcGIS software environment (Esri Inc., 2019). All stands were located on minimally sloping land (less than 15° slope), and elevation change between plot pairs was minimized. The plot pairs were spread across broad gradients of mean annual temperature (MAT) and precipitation (MAP), ranging from 0.43°C to 7.77°C and 539 to 772 mm, respectively, during the years 1961–2017 (Figure 1). Further details and data sources are provided in Eckdahl, Kristensen, and Metcalfe (2022).

### 2.2 Vegetation Survey

The 50 burnt plot overstories were largely dominated by Scots pine (*Pinus sylvestris*) with a percentage of Norway spruce (*Picea abies*) stems between 25% and 50% in five plots, between 50% and 75% in three plots, and greater than 75% in two plots. Birch stems (*Betula pendula* and *Betula pubescens*) were less than 25% in 44 plots and between 25% and 50% in six plots, of which only one was spruce dominant. All plots showed some visible charring of tree boles, though only three plots had greater than 1% plot‐wide canopy blackening. The observed lack of tree charring led to the assumption that the majority of fire impact on C stocks was restricted to the soil layers, as discussed further in Eckdahl, Kristensen, and Metcalfe (2022). Understory was nearly completely consumed in burnt plots and its prefire properties were therefore estimated by the paired control plots. There, understory was typically below 1 m in height, preventing its function as ladder fuel to pine canopies that began at several meters' height, and composed less than 2% of the C found in the soil when averaged across the 50 control plots. Adding this C pool to C loss estimates did not affect the significance of the results of this study and was therefore ignored for simplicity.

### 2.3 Soil Sampling and Measurement

Soil sampling and measurements were performed approximately 1 year postfire over the dates 5 August to 20 August in 2019. This delay was intended to allow soil temperature to stabilize and to capture the more rapid fluctuations in BC stocks due to aerosol deposition and initial eluviation but avoid discrepancies between burnt and control plots due to longer term controls on decomposition and physical transport of BC stocks (Bellè et al., 2021). Samples were taken from the mineral, duff, moss/litter soil layers from all plots (as described below). Char layer samples were additionally collected from the pyrogenic material on the surface of the burnt plots. The organic layer was considered the grouping of the duff, moss/litter and char layers while the total soil layer was considered to be the organic and mineral layers combined.

The thickness of each layer was measured in millimeters at 20 separate points equally spaced across each plot diagonal (Kristensen et al., 2015). The mineral layer was measured from its upper interface with the organic layer to its highest rock obtrusion. The duff layer was considered the conglomerate of the F (partially decomposed material) and H (humic material) layers in accordance with the Canadian system of soil classification (Canadian Agricultural Services Coordinating Committee, 1998). The moss/litter layer was all unburnt material on top of the duff layer, including visually identifiable detritus and living moss. In all burnt sites, a layer of conglomerated char formed a clear boundary on top of the moss/litter, allowing for distinct measurement. Here, char is defined as fully blackened, brittle material with apparent high heat exposure due to fire.

Soil depth in all 100 plots ranged from 8.73 to 44.71 cm and were podzols, except 6 which had no mineral soil across the measured transects and had organic layers directly on top of bedrock. The podzols had an organic (average 45.5% C by weight in all control plots, 11.5 cm average depth) layer clearly separated from a coarse grained low C (average 5.3% in control plots, 6.26 cm average depth) mineral layer below. Lacking differentiation by soil class, the variation in soil properties important to this study is expected to be best captured within the variables TEM, total C and total mass.

Samples were acquired for all four soil layers. Four mineral soil samples were taken using a 3 cm diameter, 40 cm long gouge auger corer at four corners of a square each 15 m from the plot center. Where feasible, at least 10 cm vertical mineral cores were taken; however in shallower layers a minimum depth of 5 cm each was collected. Duff samples were collected at the same plot corners as the mineral cores by excavating four soil volumes of approximately 25 × 25 cm^−2^ area and at least the full depth of the organic layer. This volume was trimmed to discard the mineral and moss/litter layers off the bottom and top of the volumes, respectively. Right angles were then gently cut with sharp scissors, and the three dimensions were measured in millimeters (collected samples were at least 400 cm^3^ each and aimed to sample the entire in situ depth). Duff and mineral soils were kept frozen until portions were freeze‐dried for separate analysis. Moss/litter samples were collected by cutting squares, with attention to preservation of the natural in situ volume, until filling a 553 cm^3^ steel container. Char layer samples were similarly collected in a 112 cm^3^ container. At least one sample each of moss/litter and char were acquired from each plot quadrant, though more were taken at equal spacing along a transect to fill the containers if the layer was thin. On the upper surface of the char layer were small portions of dry, unburnt material, which were likely postfire additions of litter to the forest floor. This material was discarded from the char collection and was not included in BC stock estimates.

The mineral layer was sieved to 2 mm with the fine fraction used for BC analysis and the coarse fraction assumed to have negligible BC. The duff layer was sieved to 4 mm with the fine fraction pulverized and analyzed for BC while the coarse fraction (mostly composed of roots) was assumed to have negligible BC content. The moss/litter and char layers were separately pulverized and analyzed for BC in their entirety.

Additional details on sampling and soil measurements are provided in Eckdahl, Kristensen, and Metcalfe (2022).

### 2.4 Black Carbon Quantification

The only data provided in addition to Eckdahl, Kristensen, and Metcalfe (2022) by the current paper was the quantification of BC in all soil layers which is found in the repository provided by Eckdahl, Rodriguez, et al. (2022). BC was quantified using CTO‐375 (Gustafsson & Gschwend, 1997; Gustafsson et al., 2001; Elmquist et al., 2004), with adaptation for soil samples (Agarwal & Bucheli, 2011). CTO‐375 was chosen to isolate an oxidation resistant portion of the PyC pool in order to directly address the effects of fire on C storage. The method also has a relatively low false positive rate, well‐tested precision and is practical for applying to a large number of samples (Hammes et al., 2007). However, by selecting for compounds of low thermolability, the method is expected to give a conservative view of overall C compound change due to the varied heating conditions of wildfire (Hammes et al., 2007). Although, recent global analysis suggests CTO‐375 produces similar PyC concentrations in soils as other common quantification methods (Reisser et al., 2016).

Samples of 15–20 mg were weighed into silver capsules (precombusted at 450°C). Thermal treatment was performed on the samples in a horizontal tube furnace (Entech EFT30‐50) at 375°C with forced airflow of 250–300 mL min^−1^ for 24 hr. The temperature was continuously monitored with an external probe and ramped slowly to prevent overshoot. After, inorganic carbon was removed by acid fumigation with 12 M hydrochloric acid in a desiccator for 4 hr. The samples were moistened with 25 μL deionized water before and after fumigation. The samples were dried on a hot plate at 60°C–70°C before measurement of the remaining C.

Total C in the remaining sample material was measured using an elemental analyzer (COSTECH ECS4010). The measured remaining C weight was divided by original sample weight to determine the weight ratio of BC (BC:W) within the plot soil layer (given in units of g kg^−1^). The performance of the elemental analyzer was calibrated every 10 samples using acetanilide (Elemental microanalysis, UK) and compared to measurements of a soil standard (Boden standard, Säntis analytical AG, Switzerland). The detection limit was 1.3 μg C, estimated as the average C response in blank runs plus five times the standard deviation (*n* = 5). No standard for BC is available, and the BC quantification was evaluated against replicated measurements (*n* = 5) of reference materials NIST1944 (sediment) and SRM 2795 (diesel soot), with published BC quantification. The measured BC concentrations in the reference materials were 0.73 ± 0.09% for NIST1944 and 67.5 ± 0.9% for SRM 2795, and are within the ranges of published values (NIST1944: 0.8 ± 0.02%, SRM 2795: 68.2 ± 0.9%) (*r*. Gustafsson et al., 2001).

### 2.5 Data Analysis

Data analysis was performed using Python 3 and the packages NumPy (Harris et al., 2020) and pandas (Wes McKinney, 2010). For all soil layers total mass, C stock calculations, bulk density, layer depth, MAT, MAP, TEM, and the time‐of‐fire drought conditions using the standardized precipitation‐evapotranspiration index (SPEI) were taken from Eckdahl, Kristensen, and Metcalfe (2022). BC for each soil layer was calculated by multiplying the derived BC:W ratio by total layer mass. BC:C was calculated by dividing total layer g BC by its total kg C. All variables formed by burnt and control plots differences were calculated by subtracting control plot values of the variable from those of its burnt pair thereby forming a single distribution. These distributions were approximated as normal and all confidence intervals were constructed at the 95% level, unless otherwise noted, using the formula

(1) $$I=\bar{x}\pm z\cdot \frac{\sigma }{\sqrt{n}}$$

where $\bar{x}$ is the sample mean, *z* is always 1.96 for the 95% interval, *σ* its standard deviation and *n* the sample size. Significance of differences between control and burnt plots was deemed to be when their interval did not include zero. The coefficient of variation (CV) of a distribution was calculated by dividing its standard deviation by its mean.

Two burnt plots and three control plots did not have mineral layers and sample size was reduced accordingly for BC:W and BC:C distributions when analyzing these layers alone. However, total BC in these five mineral layers (and one additional plot that had no mineral layer across measured transects) was set to 0 when incorporated into total soil calculations of BC:W and BC:C. One char layer sample was lost before BC analysis and therefore BC:W and BC:C for the char layers included only 49 samples. Total char layer BC for this missing sample was calculated using the average BC:W from the remaining 49 char layer samples.

All regression analyses used the ordinary least squares approach to estimate a function for a single response variable based on linear combinations of the predictor variables and an intercept term. Simple regression was performed in Python 3 using the stats.linregress method from SciPy (Virtanen et al., 2020) providing significance (*p*), correlation (*r*), and slope (*b*). Multiple regression was carried out with the OLS class in the Python 3 statsmodels package (Seabold & Perktold, 2010) with models evaluated in order of increasing Akaike information criterion (Akaike, 1974). Standardized regression coefficients (*β*) were produced by normalizing all variables (converting to *z* scores) before regression. Here, variables are considered significantly correlated when *p* values from simple regression are less than 0.05.

## 3 Results

### 3.1 Black Carbon Stock Changes Due To Fire

Quantitative results for fire‐induced changes in soil BC properties described in this section are found in Table 1.

**Table 1:** Mean Values of Distributions Formed by Subtracting Control Plot Variables From Their Paired Burnt Plots Are Given Followed by Their 95% Confidence Intervals

	BC (g BC m^−2^)	BC:C (g kg^−1^)	BC:W (g kg^−1^)
Soil	**11.6** **±** **10.4 (49.5)**	**3.75** **±** **2.76 (76.8)**	**0.172** **±** **0.157 (23.8)**
Organic	−0.0498 ± 2.3554 (−0.438)	**0.981** **±** **0.831 (37.2)**	**0.292** **±** **0.180 (24.4)**
Char	**3.94** **±** **0.83 (−)**	–	–
Moss/Litter	**−1.93** **±** **0.88 (−75.8)**	−0.278 ± 0.642 (−8.54)	−0.0834 ± 0.3236 (−5.55)
Duff	**−2.05** **±** **2.04 (−23.3)**	1.02 ± 1.19 (40.7)	0.151 ± 0.174 (13.4)
Mineral	**11.6** **±** **10.2 (96.6)**	**12.8** **±** **8.39 (85.9)**	**0.161** **±** **0.102 (42.5)**

*Note.* The percentage change calculated from the overall separate means of control and burnt plots follows in parentheses. Statistically significant fire‐induced changes are in bold.

#### 3.1.1 Total Black Carbon

**Figure 2:**
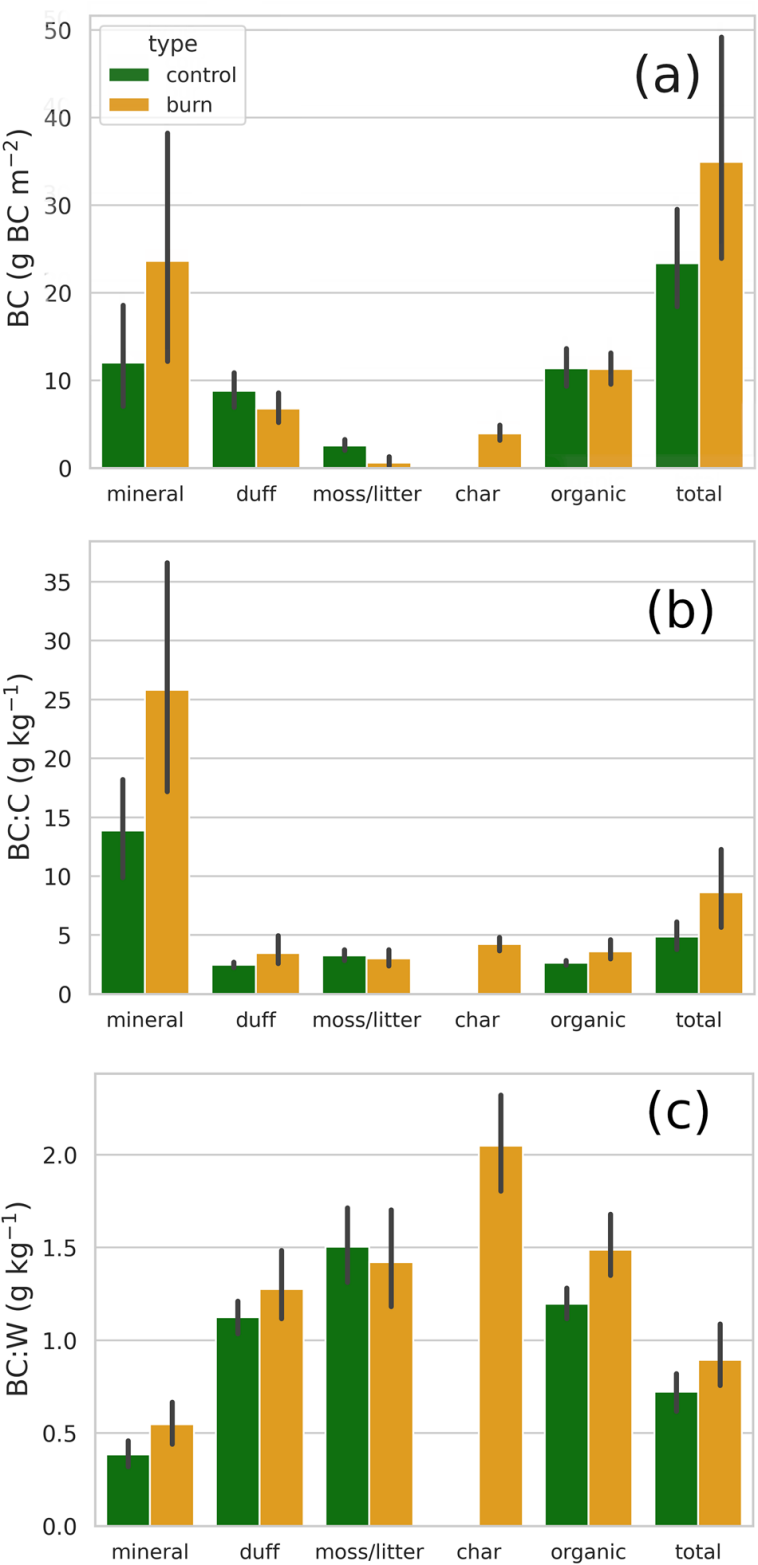
Mean black carbon (BC) stocks (a), BC:C (b), and BC:W(c) between burnt and control plots amongst forest soil compartments. The organic layer is considered the grouping of the duff, moss/litter, and char layers while the total category is the grouping of the organic and mineral soil layers. Error bars are the bootstrapped 95% confidence interval of the mean (*n* = 1,000).

Total BC was significantly lower overall in both the duff and moss/litter layers in fire plots compared to their paired controls (Figure 2a). However, including BC in the char layer resulted in no significant difference in the total organic layer BC stock as a whole between control and burnt plots. The total mineral layer BC in burnt plots was nearly double that in control plot mineral layers, resulting in an overall significant increase in burnt plot BC stocks of 49.5% within the sampled soil as a whole.

The larger error bars in Figure 2a for mineral BC stock estimates compared to organic are contributed to the overall greater variation in mineral soil mass (burnt: CV = 1.19, control: CV = 1.19) and BC:W (burnt: CV = 0.735, control: CV = 0.661) compared to organic layer mass (burnt: CV = 0.485, control: CV = 0.542) and BC:W (burnt: CV = 0.735, control: CV = 0.661). Mineral layer depth, bulk density and total mass were not significantly different across burnt and control plot pairs suggesting good sample matching. Variation in mineral BC shifts due to fire (Table 1) are attributable to the large range fire‐induced BC:W adjustments and potential plot pair mismatch in estimating prefire BC:W in burnt plots, with the latter variation assumed to be distributed randomly and directly mitigated, in terms of the 50 plot averages, by the large sample size of the study.

#### 3.1.2 Mass Ratio of Black Carbon to Total Soil and Soil Carbon

Fire did not significantly affect BC:C or BC:W in the duff and moss/litter layers, but grouped with the char layer it increased organic layer BC:C and BC:W significantly (Figures 2b and 2c). Large, significant BC increases in the mineral layer were aligned with a significant increase of BC:C and BC:W in the soil as a whole, in burnt plots relative to control. Fire‐induced increases of BC:C and BC:W were pronounced in the mineral layer, where BC:C nearly doubled in mean value. BC:W values had a range of 0.082–1.15 g kg^−1^ and 0.619–2.25 g kg^−1^ in control plot mineral and organic layers, respectively. Burnt plot mineral and organic layers have respective ranges of BC:W of 0.134–2.08 g kg^−1^ and 0.774–4.87 g kg^−1^.

#### 3.1.3 Drivers of Black Carbon Stock Changes Due To Fire

Fire‐induced mass or C loss from any layer was not related to BC differences in burnt plots relative to control in the mineral and total soil layers nor to the ratio of BC stock changes between mineral and organic layers. However, fire‐induced losses of organic layer mass were strongly negatively related to BC stock increases in burnt plot organic layers relative to control (*p* < 0.001, *r* = −0.835, *b* = −0.00139) with a weaker relation to loss of C (*p* < 0.001, *r* = −0.706, *b* = −0.00263). That is, the less organic layer material that was estimated to have burnt away the greater the amount of BC stocks in burnt plots relative to control. Strong correlations between organic layer mass and its total BC were found in burnt (*p* < 0.001, *r* = 0.750) and all (*p* < 0.001, *r* = 0.843) plots. As with losses of mass and C, lost BC from the organic layer due to fire was not significantly related to change in BC in mineral layers in burnt plots relative to control.

Organic layer losses of mass or C due to fire were not significantly related to BC:W or BC:C in the burnt plot organic layers or fire‐induced shifts of these variables. However, differences of C in burnt plot mineral layers relative to control were negatively related to fire‐induced changes in BC:C within that layer (*p* < 0.001, *r* = −0.591, *b* = −19.6 m^2^ g kg^−2^ C^−1^), that is, as C was reduced, BC:C increased between plot pairs. Organic layer C loss did not relate to BC:W or BC:C within the burnt plot mineral layers.

No measured indices of either soil drainage, time‐of‐fire drought or long‐term climatic regime (i.e., TEM, SPEI, MAT, or MAP) could be linked through regression analysis to total BC stock differences in mineral or organic soil layers between control and burnt plots or to BC stocks in burnt plots alone.

### 3.2 Black Carbon in Control Plots

Results for BC property mean values and their variation in control plot soil layers presented in this section are found in Table 2.

**Table 2:** Mean Values and 95% Confidence Intervals of the 50 Control Plot Value Distributions for Each Sampled Soil Layer's Total BC, BC:C, and BC:W

	BC (g BC m^−2^)	BC:C (g kg^−1^)	BC:W (g kg^−1^)
Soil	23.4 ± 5.8	4.88 ± 1.15	0.722 ± 0.098
Organic	11.4 ± 2.1	2.63 ± 0.22	1.20 ± 0.09
Moss/Litter	2.56 ± 0.59	3.28 ± 0.46	1.50 ± 0.21
Duff	8.80 ± 1.95	2.47 ± 0.23	1.12 ± 0.10
Mineral	12.0 ± 5.9	13.9 ± 3.9	0.392 ± 0.072

#### 3.2.1 Total Black Carbon

**Figure 3:**
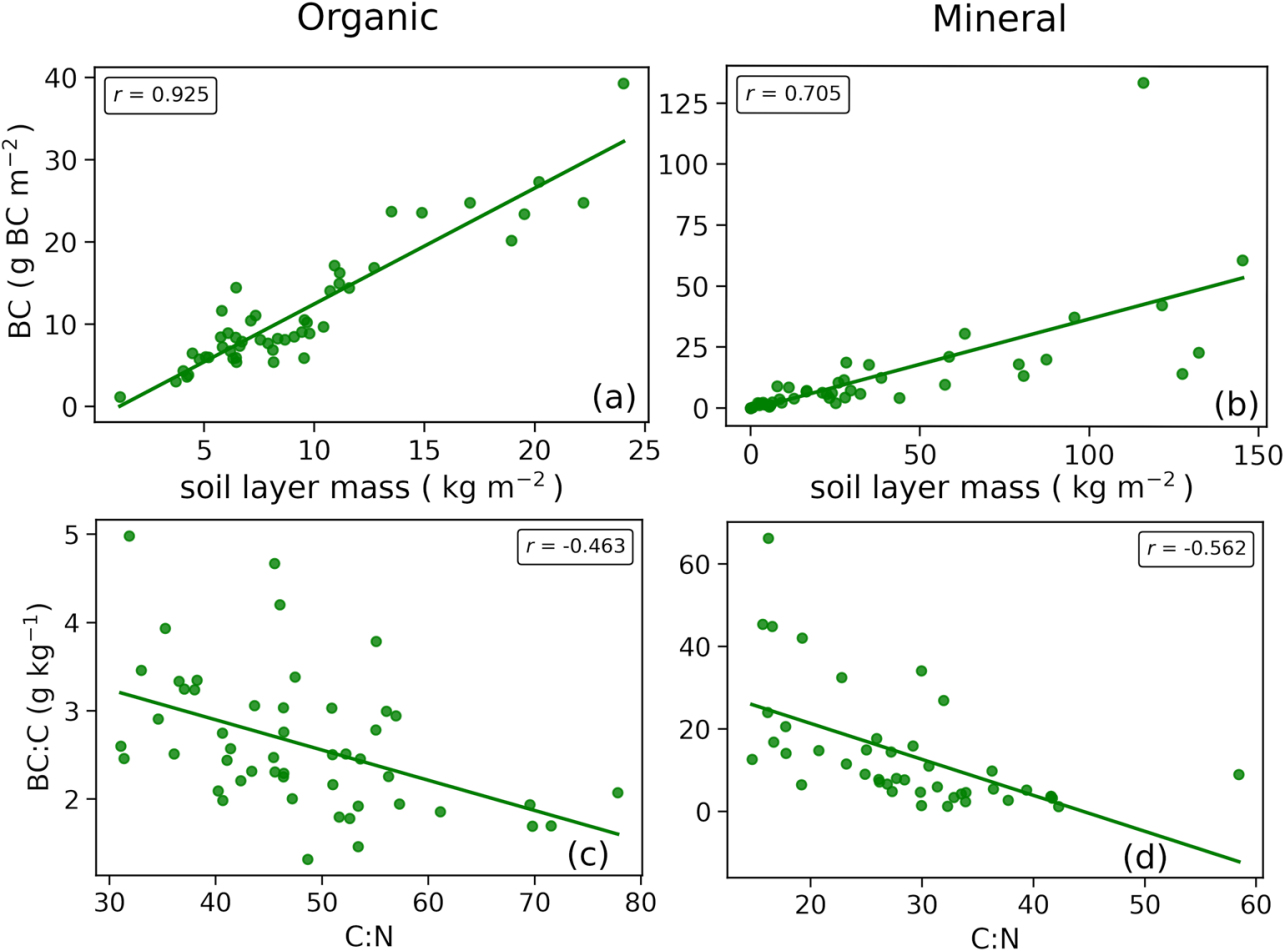
Simple regression charts for the 50 control plots regarding organic layer total black carbon (BC) against its layer mass (a), mineral layer total BC against its layer mass (b), organic layer BC:C against its C:N (c), and mineral layer total BC against its C:N (d). All regressions have *p* < 0.001.

Total mass of each respective soil layer was the strongest correlator to control plot BC stocks in the organic (*p* < 0.001, *r* = 0.925, Figure 3a), mineral (*p* < 0.001, *r* = 0.705, Figure 3b) and total soil (*p* < 0.001, *r* = 0.623). Total C was less predictive of BC stocks within organic (*p* < 0.001, *r* = 0.901), mineral (*p* < 0.001, *r* = 0.489), and total soil (*p* = 0.066, *r* = 0.262) layers.

#### 3.2.2 Weight Ratios of Black Carbon

Linearly combining the three factors used to calculate total ground‐area‐normalized BC (i.e., BC:W, soil bulk density and soil layer depth), variation in BC:W appeared to have a strong influence over BC stocks in control plots via standardized regression coefficients in the mineral, organic and total soil layers (Table 3).

**Table 3:** The Measured Variables BC:W, Bulk Density and Depth Were Linearly Combined to Explain BC Stocks (Calculated as the Product of These Variables) in Multiple Regression for the Organic, Mineral and Total Soil Layers

Layer	*p*	*R* ^2^	BC:W	Bulk density	Depth
Soil	<0.001	0.709	0.784	0.799	0.467
Organic	<0.001	0.926	0.351	0.491	0.644
Mineral	<0.001	0.652	0.491	0.179	0.655

Control plot organic layer BC:W was correlated to its C:N ratio (*p* = 0.006, *r* = −0.384). However, the C:N ratio had a slightly stronger correlation to BC:C (*p* < 0.001, *r* = −0.463, Figure 3c).

Control plot mineral layer BC:W was weakly correlated to its C:N (*p* = 0.185, *r* = −0.197) with the model being improved (*p* = 0.001, *R* ^2^ = 0.269) by combining C:N (*β* = −0.241) and MAT (*β* = 0.477) in multiple regression. BC:C had increased correlation to C:N (*p* = 0.001, *r* = −0.562, Figure 3d) with the model being enhanced (*p* < 0.001, *R* ^2^ = 0.389) by including the C:N ratio (*β* = −0.540) with MAT (*β* = −0.273). Thus the proportion of mineral soil C stored as BC increased with decreasing C:N ratio and MAT.

## 4 Discussion

### 4.1 Effects of Fire on Black Carbon Stocks

#### 4.1.1 Total Black Carbon

Averaging across the 50 burnt plots surveyed, fire increased BC stocks by 11.6 g BC m^−2^ (49.5%) nearly entirely due to an almost doubling in mineral layer BC, but had no significant effect on total organic layer BC stocks despite its substantial reduction in total mass. This addition is within an order of magnitude of recent estimates of total PyC addition rates for the region (Jones et al., 2019), assuming an approximate 100 years fire return interval. Sampling over a broader spectrum of heat‐altered compounds could have produced substantially larger estimates of total PyC production in these forests (Schmidt et al., 2001). However, recent global analysis of available soil PyC stock data in literature suggests CTO‐375 tends to measure similar amounts of PyC concentrations in soil as other common quantification methods (Reisser et al., 2016).

The larger concentration of fuel (i.e., combustible organic material) and proximity to an oxygen source (the atmosphere) should have allowed for more intense burning conditions in the organic layer, however the mineral soil below may have received enough heat (along with a lowered oxygen supply) for production of BC in situ. Although, mineral soil temperatures have been observed to be low in boreal forest fire and so the most likely explanation for the enhanced BC stock in the mineral soil observed in this study is that fire caused a pulse of material with high BC:C to percolate from upper soil layers into the mineral layer within the 1 year postfire period (Bellè et al., 2021; Santín et al., 2015). This preferential and rapid transport of BC relative to other C forms from organic layers may indicate alteration of their prefire BC pool due to fire (e.g., production of new BC or chemical change of prefire BC) or due to soil restructuring that released older, bound BC. Further, controlled studies are required to determine the mechanisms of BC production and mobilization in similar systems, however it is clear from these results that explicit consideration and inclusion of the mineral layer was critical for accurate estimation of the impacts of fire on soil BC stocks in the sampled Fennoscandian systems. Forests with analogous soil structure (separated high C organic, low C mineral) and fire propagation (ground fire), which are common across the broader boreal region, have the potential to exhibit similar importance of mineral layer BC, and therefore further attention to this pool is merited.

#### 4.1.2 Ratio of Black Carbon to Total Carbon

The mineral, organic and total soil all had significantly increased BC:C in burnt plots relative to control. The increase of BC relative to C along with increases in total soil bulk density in the organic layer may have significant effects on ecosystem functioning. Particularly, increased chemical recalcitrance of the C pool along with lowered oxygen availability due to the observed compaction of the soil structure in burnt plots (Eckdahl, Kristensen, & Metcalfe, 2022) may reduce efficiency of decomposition and nutrient cycling (Tan & Chang, 2007). In contrast, increased volumetric concentration of PyC may provide more suitable conditions for soil life through improved habitat structure, water retention and slow release of adsorbed fire‐mineralized nutrients (Makoto et al., 2011, 2012; Warnock et al., 2007). However, organic layer BC:C only increased from 0.236% to 0.362% due to fire which may not be a sufficient change to play a significant role in the overall bioavailability of soil C. Also, BC separated using the CTO‐375 method is generally considered to be soot‐like with a relatively minor ecosystem effect relative to other PyC forms (Elmquist et al., 2006; Masiello, 2004). Although, its occurrence may covary with other, more biogeochemically active portions of the PyC spectrum, so BC as measured by CTO‐375 could serve as a useful proxy for the effect of fire‐induced fuel transformation on ecosystem function.

#### 4.1.3 Black Carbon Relationship to Environmental Variables and Fire Severity

Mean annual temperature, MAP, SPEI, and TEM did not explain differences in BC stocks between control and burnt plots nor patterns within burnt plots alone. However, some statistical signals were apparent when examining fire severity and changes in soil layer mass.

Burn‐control plot pair differences of C and BC:C were negatively correlated in mineral layers, suggesting a portion of their C pool may have been converted to BC during fire. Because C stocks were not significantly different in burnt and control mineral layers and BC pools were only 1%–3% of total mineral C, this signal is considered weak and may not represent a consistent trend.

On the other hand, fire‐associated organic layer estimated losses of both total mass and C were negatively correlated to increased burnt plot organic layer BC relative to control. Although, insignificant differences of total organic layer BC and a lack of correlation between fire severity and BC:C within this layer make it difficult to disentangle an innately fire‐driven variation on total BC stocks when they are also predicted so strongly by total mass of the soil layer material. However, the significant overall increase in BC:C in the organic layer shows a clear fire‐induced effect on its C pool.

Finally, within the entire soil profile, BC gain did not correlate with total soil C loss. Additionally, the mean value for the ratio of BC gain to C loss, which is a ratio often applied to C emission estimates to predict BC production (Jones et al., 2019), had high error (4.35 ± 15.6 g kg^−1^ at 95% confidence). This ratio has largely been developed to reflect variation in vegetation and surface fuel cover and their associated flammability and is not well tested in deeper burning ground fire‐dominated systems. It is proposed that C losses due to ground fire are likely not predictive of soil BC additions, at least at the 1 year postfire stage in boreal forests similar to those in Fennoscandia. Predicting total BC production in ground fire‐dominated systems may require more site‐level details on soil material source and structure and their effect on heat and gas exchange, which are parameters known to heavily influence PyC formation (Preston & Schmidt, 2006).

### 4.2 Black Carbon Stocks in Control Plots

The average total soil BC stock over the 50 control plots was estimated as 23.4 ± 5.8 g BC m^2^ (4.56 g BC kg^−1^ C^−1^). On average, approximately half of this total soil pool was found in the mineral layer with the other half in the organic layer despite the organic layer having over 4 times the amount of total C in control plots. This pattern is congruent with an over 5‐fold larger BC:C in mineral relative to organic layers in control plots (Figure 2b). Few estimates of BC soil storage are available in boreal Eurasia (there is a severe deficit within high latitudes in general), of which none are in Fennoscandia. However, recent model upscaling to Fennoscandia suggests the range of PyC in its soils is 1,470–4,010 g PyC m^−2^ with 44–105 g kg^−1^ PyC:C (Reisser et al., 2016), where the C pool is derived from modeled global estimates of soil organic C. These PyC storage estimates differ drastically from those measured for BC in the current study despite its measured BC input rates agreeing well with current global estimation methodology. This sizable discrepancy further highlights research deficiency regarding PyC removal rates (Bowring et al., 2022), sampling in diverse ecosystem types (Coppola et al., 2022; Reisser et al., 2016), and suitability of upscaling covariates (Reisser et al., 2016). Furthermore, the current study demonstrates the strong effect of time‐since‐fire on sampling, where soil BC stocks are not unlikely to be at least 50% higher in plots that are recently burnt.

Because the 2018 fires appeared to produce no significant increase in organic layer BC at only 1 year postfire, the total build up of BC in control plot organic layers seems likely to have originated from previous direct exposure to fires under different conditions than those in 2018 or from extended deposition of aerosol BC from separate wildfires or anthropogenic sources (Coppola et al., 2022). Because the moss/litter layer is presumably made up of newer, non‐fire‐exposed additions of organic matter than the duff layer below, its higher BC:C in control plots compared to burnt is suggestive of significant BC additions from these remote sources.

The strongest predictor for control plot BC stocks in organic, mineral and total soil layers was their respective total mass while MAT, MAP, TEM and C:N were unrelated. While it may seem obvious that total BC should strongly correlate to its layer's mass since BC is calculated by the mass multiplied by BC:W, maximum and minimum BC:W in organic and mineral layers are separated by at least an order of magnitude providing ample flexibility for BC:W to vary to counteract the observed relationship. Hence, it is interpreted that the strong correlation between BC stocks and total soil layer material mass represents at least in large part a genuine biogeochemical trend rather than simply a statistical artifact of the BC calculation method. The enhanced correlation of soil mass over soil C suggests it may in some situations provide a better predictive measure of PyC stocks than the currently used soil organic C (Reisser et al., 2016). Although, this relationship may depend on the mobility or decomposability of a particular PyC fraction and its input rates relative to inputs of non‐pyrogenic C to the soil.

BC:C, however, correlated negatively with C:N in organic layers and both C:N plus MAT in mineral layers. Assuming a reduced C:N is indicative of enhanced soil decomposition (Callesen et al., 2007), the negative correlation between organic layer BC:C and C:N in control plots suggests BC is resistant to decomposition in these soils. This preservation of BC relative to total C during soil decomposition may be due to its enhanced oxidation resistance and interaction with soil structure (e.g., enhanced mineral adsorption affinity) of BC (Preston & Schmidt, 2006).

### 4.3 Black Carbon Equilibrium Storage and Removal Mechanisms

The unexpected lack of fire effects on organic layer total BC at only 1 year postfire and the approximate doubling of BC stocks in burnt plot mineral layers raises important questions about the future trajectory of BC in the sampled ecosystems.

First, will burnt plot BC stocks return to near control plot levels before the next fire event, or will the 2018 fires have caused a substantial net increase of BC soil stores? Soils may have a maximum sorption potential for BC which is mainly dependent upon the amount of soil and its structure (Abramoff et al., 2021; Georgiou et al., 2021). The strongest predictor for control plot BC stocks in organic and mineral layers was their respective total mass with no significant contribution from climate, soil drainage and soil decomposition state (i.e., MAT, MAP, TEM, and C:N), though BC:C correlated negatively with C:N in organic layers with an additional effect of MAT in mineral layers. Fire‐induced loss of organic layer mass was strongly negatively correlated to BC addition within the burnt organic layers, while control plot organic layer mass had an even stronger relationship to its total BC. Therefore, BC storage may be near a maximum stable storage potential in these systems that is most strongly controlled by the amount of soil material present to hold these BC stores, while variation in climate and soil characteristics (i.e., C:N) appear to have only small qualitative effects on the BC pool (i.e., BC:C). When inputs exceed this maximum storage potential, BC loss may begin to be more controlled by the balance of factors determining BC input rates (e.g., fire return interval and active fire properties such as intensity) versus output rates (e.g., precipitation patterns, soil drainage, topography, and soil biological activity).

Second, how and from what portion of the soil layer will BC stocks be removed? If control plot soil compartments represent maximum BC storage potentials of their burnt pairs, larger removal of BC from the mineral layer is expected with minimal removal from the organic layer. Additionally, without some process(es) accounting for significant removal of BC from burnt plot mineral layers, the 2018 fires would have added an amount of mineral BC equivalent to what has been accrued throughout the entire pre‐2018 fire history of the forests. BC stocks appear to be selectively preserved in the organic layer when compared to overall C under fire conditions (increased BC:C) and decomposition processes (BC:C negatively correlated to C:N). Because boreal mineral layers tend to be less bioactive overall relative to the organic layer, due to low overall C availability in subsoils, biological BC decomposition is expected to be small compared to that in organic layers (Karhu et al., 2010). Temperature measurements have also found minimal heating of the mineral layers during fire (Santín et al., 2015). Therefore, the majority of the substantial BC loss from the mineral layer needed to offset the apparently high rates of fire‐associated input is likely to occur through vertical or lateral water transport to deeper stores and waterways.

Lastly, what controls the rate of BC removal? Initial BC removal rates in the fire‐affected mineral layer may be enhanced by higher BC concentration and an initial flushing of new, more soluble and suspendable forms, but decline as this highly leachable portion is lost (Bellè et al., 2021) leaving behind fractions that may require additional processing to mobilize (e.g., physical degradation (Coppola et al., 2022)). However, solubility of highly aromatic PyC has been observed to increase with age which has been related to oxidative functionalization of aromatic rings with polar hydroxyl and carbonyl groups, though this functionalization can also be related to the strength with which PyC forms coordination compounds with mineral grains (Coppola et al., 2022; Preston & Schmidt, 2006; Santín et al., 2016). Therefore, this stage of leaching may be controlled by the proximity to the sorption potential of the soil. These two counterbalancing properties of PyC solubility and sorption affinity may account for the consistent ratios of BC to total C being found in waterways over time, despite boreal wildfire burnt area in individual catchment areas being concentrated within fire seasons that are generally separated by several years (Ding et al., 2015; Jaffé et al., 2013; Wagner et al., 2015).

**Figure 4:**
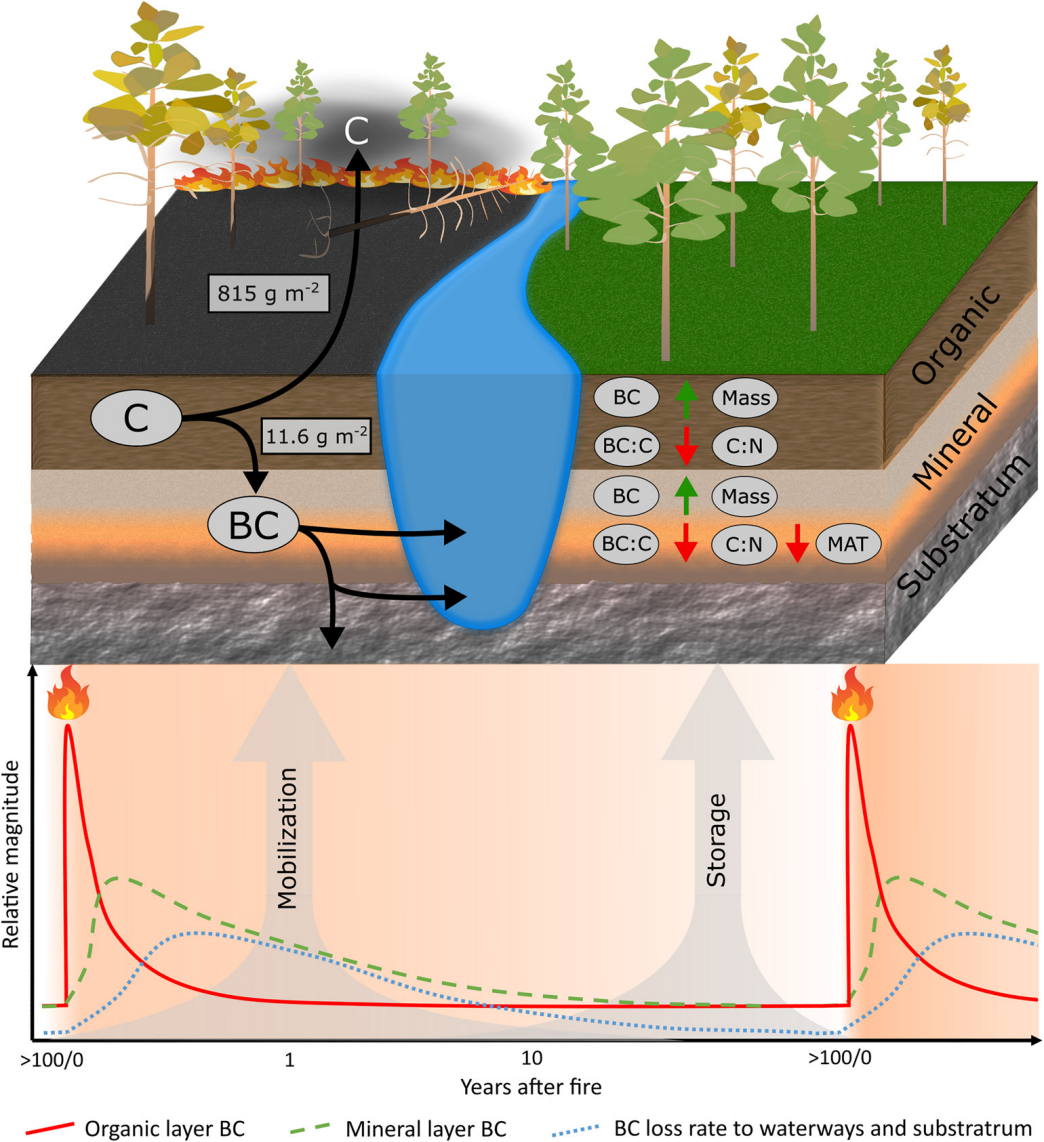
Conceptual diagram of carbon (C) and black carbon (BC) cycling proposed by the results of this study. The landscape diagram depicts temporal division of early postfire BC synthesis and mobilization (left) and late postfire BC storage patterns (right). Measuring the 50 plot pairs, fire was estimated to release an average of 815 g C m^−2^ from the soil to the atmosphere while adding 11.6 g BC m^−2^ to the mineral layer which is assumed to have percolated from the organic layer above over the 1 year postfire period. This high mobility of BC and its strong correlation to total soil layer mass in late postfire control plots suggest this fraction of the pyrogenic C spectrum depends largely on adsorption sites to remain within the soil profile. Negative correlation of soil layer BC:C to the C to nitrogen ratio (C:N) suggests BC is relatively resistant to decomposition processes compared to the larger C pool. Mineral layer BC:C has an additional negative effect of mean annual temperature. This resistance, along with low overall decomposition in mineral layers and the measured approximate doubling of its BC stocks in recently burnt forests, indicates a large portion of the BC additions are lost from this layer to waterways and deeper subsoils over the fire interval. The figure's lower panel charts a hypothetical time series of BC transport, where fire induced increases of BC in the organic layer are largely released within 1 year to lower subsoils. Mineral layer BC stocks are released more gradually to waterways and the substratum until stabilizing at an amount proportional to its total mass before the return of fire.

While dissolution and transport of BC and C from soil stores may be observed to follow similar drivers or patterns (Coppola et al., 2022), evidence from the plots in this study suggests their major sources are from the mineral and organic layers, respectively, implying a spatial decoupling of their removal mechanisms. It is therefore proposed that mineral soils in particular can act as an important buffer between short‐ (collecting and storing produced BC within 1 year after fire) and long‐term (steady aqueous transport towards limnic and oceanic sedimentation) BC dynamics (Figure 4).

It would be of interest to estimate the impact that changing climate and fire regimes would have on BC in soils. Climate change is expected to have a net negative impact on boreal forest C storage, namely by preventing the buildup of organic layer stocks through a higher fire return interval (Walker et al., 2019; Whitman et al., 2019). Thus the mineral layer may play a larger future role in the sequestration and protection of the declining overall C, so improved understanding of inputs, outputs and storage capacity for different C compounds in this layer is likely to become increasingly important (Pellegrini et al., 2021).

### 4.4 Black Carbon Representativeness of the Pyrogenic Carbon Pool

The CTO‐375 method succeeded in demonstrating a clear fire response on total BC in mineral soils along with an increase in BC:C in both mineral and organic layers. Out of the commonly practiced PyC characterization methods, CTO‐375 appears to be most resistant to producing false positives (i.e., incorporating non fire‐affected C) (Chang et al., 2018; Hammes et al., 2007). Additionally, usage of high temperature (375°C) and an oxygen rich environment to isolate the most oxidation resistant portion of the soil C pool may give a more direct perspective on the thermooxidative lability of soil C in response to wildfire when compared to methods using visual or chromatographic separation of PyC which are often then subject to assumptions derived from chemical characterization based on presence of only broadly defined patterns of functional groups (Hammes et al., 2007). For these reasons CTO‐375 appears to be an effective tool for quantitative analysis of fuel transformation over broad ranges of fire and soil properties.

However, by isolating a particularly non‐thermolabile portion of the PyC spectrum, a conservative view of overall fuel transformation may have been extracted by using CTO‐375 in this study. This may be especially true in Eurasian boreal forests which have been observed to have lower intensity fire conditions than their North American counterparts (de Groot, Cantin, et al., 2013). Further, CTO‐375 does not provide direct morphological or chemical structure information that may aid in deciphering the potential mechanisms by which the resulting PyC fraction could affect soil structure and elemental cycling. Though, from the results of this study, CTO‐375 isolated PyC appears to be a highly mobile fraction, so the method may be a valuable tool for monitoring the transport of PyC to limnic and oceanic sediment, where this characterization technique has already been widely used as a quantitative tool (Elmquist et al., 2004; Masiello, 2004).

No PyC fraction is understood well enough to predict its oxidation resistance under fluctuating in situ environmental factors (e.g., regarding delivery of oxidizers, catalyzers and activation energy to chemical bonds). Moreover, little predictive knowledge is available concerning the amounts, structural forms and synergistic functional roles of the different isolatable PyC fractions formed under varied fire and ecosystem conditions. This makes it difficult to compare the effects of PyC on ecosystems where different characterization methods may produce conflicting results when generalized to the entire PyC continuum (Schmidt et al., 2001). Accordingly, PyC research may benefit from further comparative analysis of the PyC pools defined by common characterization methods and their interrelation regarding production and destruction in fire, effects on ecosystem functioning and longevity within local and global C cycles. In particular, it may be beneficial to develop organization of PyC into functional classes of intrinsic chemical recalcitrance (oxidation resistance), structural occlusion (accessibility to oxidizers) and mobility (ability to escape degrading environments), in order to more readily predict their roles in ecosystems and C storage potential.

### 4.5 Summary and Outlook

This study has served as a highly replicated, methodologically consistent survey of a refractory subset of PyC across large environmental gradients in burnt and unburnt boreal forests with attention to its position within the soil profile. Fire doubled total BC stocks in the mineral layer with no change in the organic layer, resulting in an overall significant increase in the soil as a whole. Fire severity, climate, drought level, and soil drainage could not be strongly linked to BC production or its long‐term storage, however MAT and C:N covaried negatively with BC:C in control plots. Additionally, within control plots, the mass of the organic and mineral layers correlated strongly with their respective total BC stocks. Because the majority of observed BC soil additions likely originated from organic layer material, these additions being found nearly entirely in the mineral soil 1 year after fire is suggestive of the mineral layer being an important intermediate storage pool of BC buffering its production via wildfire heating and aqueous transport out of the ecosystem. Further understanding of the mechanisms and intermediate storage pools regarding PyC production and transport is a crucial step toward linking the global fast and slow C cycles.

## Data Availability

The BC data produced specifically for this paper is found freely available in the following repository: https://doi.org/10.5281/zenodo.7010192. All additional data used for analysis in this paper was taken directly from the following publication: https://doi.org/10.5194/bg-19-2487-2022.
